# Building a New Framework for Evaluating the Livability of Living Space on the Basis of the Daily Activities of Rural Residents: A Case Study of Jianghan Plain

**DOI:** 10.3390/ijerph191710615

**Published:** 2022-08-25

**Authors:** Xiaoyue Li, Bin Yu, Jiaxing Cui, Yuanyuan Zhu

**Affiliations:** 1 College of Urban and Environmental Sciences, Central China Normal University, Wuhan 430079, China; 2 Key Laboratory for Geographical Process Analysis and Simulation of Hubei Province, Central China Normal University, Wuhan 430079, China

**Keywords:** livability of rural living space, assessment, daily activities of rural residents, Chinese rural, Jianghan Plain

## Abstract

The evaluation of the livability of rural living spaces is an important aspect of rural sustainable development, which is related to the well-being of rural residents. This study proposes a new evaluation framework for assessing the livability pattern of rural living spaces from the major components of the daily activities of rural residents. It fully considers whether the living space within a certain geographical area can meet the needs of rural residents in terms of residence, employment, consumption, leisure, and other types of daily activities. This study also establishes a comprehensive index system that includes 36 indicators and develops a comprehensive assessment method for evaluating the livability pattern of rural living spaces. Moreover, this research conducts a case study on the spatial pattern of rural living spaces in China’s Jianghan Plain in 2018. We determine that the livability of rural living spaces in Jianghan Plain exhibits an evident “grade difference” characteristic. The overall livability level is not high. Seven problem counties from three categories are delineated on the basis of the score status. The new framework and conclusions of this study are conducive to the future targeted construction of livable rural living spaces.

## 1. Introduction

Since its reform and opening up in 1978, China has experienced rapid urbanization and industrialization [1]. According to the websites of the Central People’s Government of China and the National Bureau of Statistics of China, the urbanization rate of China’s resident population increased from 17.92% in 1978 to 60.60% in 2019. Nevertheless, 509.79 million people were still living in rural areas by the end of 2019. Therefore, the development of China’s rural areas cannot be disregarded. Moreover, rapid industrialization and urbanization have triggered dramatic spatial changes in the countryside, resulting in dramatic geographical changes and unparalleled achievements in rural development [2].

However, there are some problems with rural development in China. Examples include the prominent contradiction between industry and agriculture, the imbalance between urban and rural areas, and the deterioration of the rural living environment [3]. Individual rural areas are alienated into “super villages”, some rural areas are degraded into “hollow villages”, and more rural areas are experiencing unprecedented difficulties in their development [4]. In 2017, the central government of China proposed the Rural Vitalization Strategy (RVS) in the report of the Nineteenth National Congress of the Communist Party of China [5]. The RVS proposes the general requirements of prosperous industry, ecological livability, civilized countryside, effective governance, and affluent living. Moreover, the RVS points out that ecological livability is the key to the rural revitalization strategy and that the improvement of rural habitats should be strengthened [6]. Building a more livable rural area is the major component of rural development. Rural livability evaluation is the premise and foundation of improving the level of rural living environments [7]. Human decision making and rural public policy influence the livability of rural settlements [8,9]. Rural living space is a space for the living activities of rural residents and the spatial foundation of rural regional development. Measuring and analyzing the livability level of rural living spaces are important elements of rural geographical research in the new era. These important elements need to be considered when exploring how to improve the well-being of rural residents and the sustainable development of rural areas in the context of the new era.

In the process of rapid industrialization and urbanization, people are beginning to make comprehensive judgments and reflections on the livability of areas. The study of urban livability has received considerable attention, and related studies include urban livability, the livability of living space, and the perception of urban livability [10,11,12]. The concept of rural livability was introduced to rural studies during the 1950 s against modernization and urbanization, i.e., a reflection of counter-urbanization [13]. With the gradual implementation of China’s rural development strategy, along with the transformation of rural socioeconomic characteristics, rural living spaces are undergoing a dramatic reconstruction. Moreover, rural research has expanded from geography to multiple disciplines [14]. Relevant studies include the planning and practice of ecological landscapes and infrastructure construction in livable rural areas. Such studies also include identifying the influences of location conditions, human behavior, settlement evolution, spatial patterns, and settlement locations on the construction of rural livability [14,15,16,17].

Overall, the academic community has achieved a series of results regarding livability by focusing on the degree of environmental excellence or the level of the livability of residents’ living conditions. Research based on using the major daily activities of rural residents to analyze the degree of the livability of the living spaces of rural residents remains insufficient. These studies tend to focus on macroscopic issues, such as rural planning, environment, and transformation. However, research on the microscopic subjects of rural habitats, i.e., rural residents, has been neglected. As the economy and society develop, residents start to pursue the comfort of living with higher quality [18]. With the development of rural areas, people’s needs for a better life have become increasingly broad, and China’s rural development should give more attention to the needs of residents [19]. The state of the rural residential environment is ultimately the result of human behavior. In addition, the behavioral patterns of microscopic subjects and the factors that influence their behavior should be understood. With the acceleration of urbanization, human activities have played key roles in the development of livable villages [20,21]. However, considering the availability of rural data and other reasons, research on the livability of rural living spaces should be further advanced. For example, existing studies lack consideration of the daily activities of rural residents despite the livability of rural living spaces being closely related to their behavior [22].

Therefore, we propose a new framework for assessing the livability patterns of rural living spaces. This framework is based on the major elements of rural residents’ daily life activities. The evaluation of the livability of rural living spaces is divided into four dimensions: residential, employment, consumption, and leisure activities. This framework comprehensively and objectively considers the ability of living space within a certain geographical area to meet the needs of rural residents for their daily activities. The framework also comprehensively utilizes remote sensing, traditional statistical, and geospatial big data to assess the livability of rural living spaces.

In accordance with the evaluation model of the livability of rural living spaces constructed from the perspective of the daily activities of rural residents, the overall and dimensional levels of the livability of living space in the Jianghan Plain, the most well-developed traditionally agricultural area of rural living spaces in China, is calculated as a case site. This study produces a spatial pattern map of the livability of rural living spaces in Jianghan Plain. The study then grasps the overall level of the livability of rural living spaces and the multidimensional characteristics of the livability structure of the region. Finally, this study identifies the areas with problems in the development of livability and the shortcomings of livability in these areas. This study contributes to an understanding of the connotation of the livability of rural living spaces, provides theoretical support and a practical basis for the improvement of the livability of rural living spaces and the construction of livable rural areas in Jianghan Plain, and may also provide a practical case reference for other similar areas.

## 2. Materials and Methods

### 2.1. Study Area

**Figure 1:**
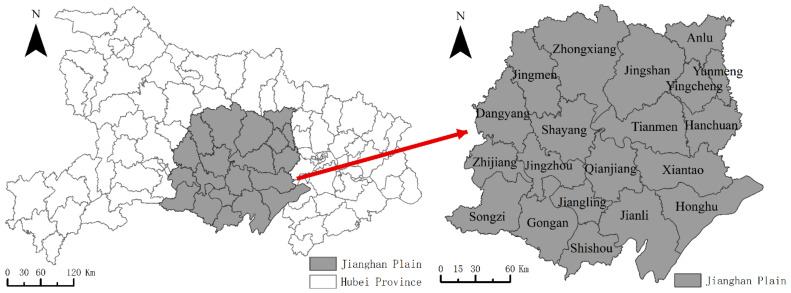
Location of the study area.

Jianghan Plain (Figure 1) is located in the central region of China. It is a traditionally agricultural region in China, one of the important grain-producing regions and agricultural production bases of the country. With a long history of agricultural development and well-developed rural living space, the region is a typical area for the study of the livability of rural living spaces in China. Meanwhile, since the advent of the 21st century, regional urbanization has accelerated, rural spaces have changed dramatically, and rural living spaces have been greatly affected. The livability of rural living spaces is related to the production and living activities of rural households, which is of great significance to the improvement of the well-being of rural residents. Therefore, studying the livability patterns of rural living spaces in the Jianghan Plain of China from the perspective of residents’ daily activities is urgent and necessary.

Jianghan Plain, which is located in the south-central region of Hubei Province between 111°14′–114°5′ E and 29°26′–31°36′ N, includes 20 cities, counties, and districts (all county-level administrative districts). Jianghan Plain is an alluvial plain formed by the impact of the Yangtze River and the Han River, with a flat topography. The population of the study area in 2018 was 15.4624 million, and the rural population was 6.7164 million.

### 2.2. Data Sources and Preprocessing

To analyze the case in more detail, five types of data of Jianghan Plain were obtained, namely land use, point of interest (POI), socioeconomic characteristics, administrative boundaries, and road data. Land use data were extracted from the land use data products of Hubei Province in 2018 on the website of China’s geographic national information monitoring cloud platform [23]. On the basis of Landsat’s Thematic Mapper (TM)/Enhanced TM/Operational Land Imager remote sensing images, the dataset was formed by using a remote sensing information extraction method combined with field measurement. We referred to the existing local and foreign land use/land cover classification systems and then conducted band selection and fusion, image geometric correction and registration, image enhancement, splicing, and clipping. We included 6 first-class categories, land use/land cover datasets of 25 secondary classifications, and some tertiary classifications with a resolution of 30 m. The POI information of Jianghan Plain was obtained from Gaode Maps in September 2019 by using Python. The POI data include spatial information, such as those of schools, hospitals, factories, and supermarkets, and attribute information, such as the name, longitude, and latitude of each object. Table 1 presents the POI data of the factors used to evaluate the livability level of rural living spaces in Jianghan Plain. The social and economic data were collected from the 2019 Hubei Statistical Yearbook and the 2019 Hubei Rural Statistical Yearbook. The vector data (administrative boundary) of Jianghan Plain and its county-level administrative units were derived from China’s 1:1 million basic geographic information database [24]. The level road network data were extracted from the Open-Street-Map [25,26]. In accordance with China’s “Highway Engineering Technical Standards” and “Urban Road Engineering Design Specifications”, the obtained data of railways, highways, national roads, provincial roads, county roads, township roads, first-class roads, second-class roads, third-class roads, and fourth-class roads were unified into the same road system on the basis of design speed and traffic capacity. In particular, first-, second-, third-, and fourth-class roads were classified as highways, national roads, provincial roads, and county roads, respectively. Then, the data were checked.

### 2.3. Research Framework

**Figure 2:**
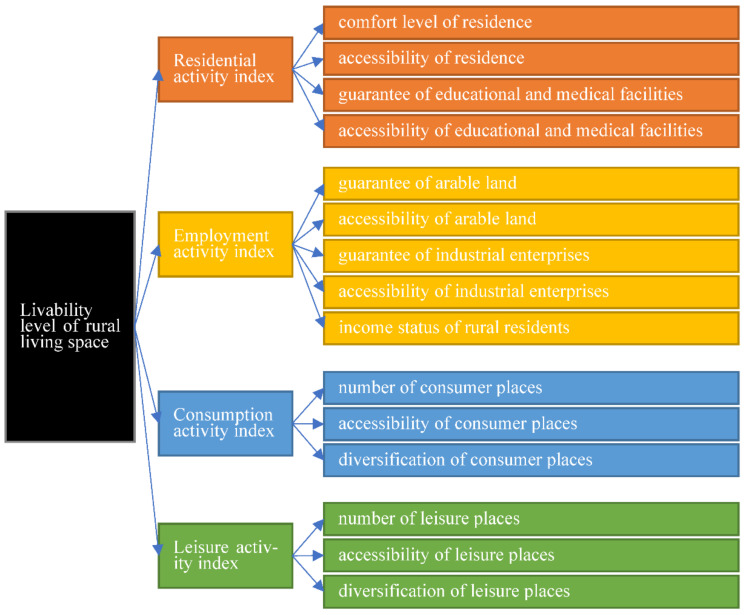
Analysis framework of the livability of living space in rural areas.

In this study, we proposed a new framework for assessing the livability pattern of rural living spaces. The livability of rural living spaces was analyzed using a comprehensive index system. Rural living space refers to a polymer formed by the overlapping of daily activities for rural residents in a certain territory, such as residence, employment, consumption, and leisure. Rural living space also refers to an organic body wherein spatial form, implication, and meaning are inherently associated with one another [14,27]. Livability is the satisfaction state of people’s behavior and demand for social civilization, economic prosperity, a beautiful environment, resource bearing, low-cost living, and public safety in a certain area. Its essence is that people must meet all types of behavior and needs in life [15]. The livability of living space in rural areas refers to the characteristics of living space that can meet the needs of rural residents in various daily life activities [28]. Rural livability has received increasing attention in various fields. At present, however, no consensus has been reached with regard to the definition and measurement strategy for the livability of the living space in rural areas in the available literature [29]. In this context, the livability of living space in rural areas is defined in the current study as “the ability of living space to meet the needs of rural residents’ daily activities, such as residence, employment, consumption, and leisure within a certain geographical range”. Figure 2 shows the analysis framework of the livability of living space in rural areas.

From the perspective of rural residents’ daily activities, living activities are the most basic needs of the daily activities of rural residents. Moreover, the spatial livability of the residential activity dimension is the basic premise of the livability level of rural living spaces. Rural residential area per capita and the degree of greening around a residence were selected to represent the comfort level of a residence. In addition, road length per unit area was chosen to represent the accessibility of a residence [30,31]. The number and accessibility of education and medical facilities in a residential area were designated to characterize the guaranteed degree and accessibility of educational and medical facilities around the residence.

Employment activities provide the basic material guarantee for the normal development of daily activities in rural areas. Moreover, the livability of rural spaces at the level of employment activities is a material prerequisite for the level of the livability of rural living spaces. The level of livability under the dimension of employment activities refers to the production activities of rural residents engaged in agricultural and non-agricultural activities in the surrounding areas, the degree of transportation convenience, and the status of output income. The indicators that describe the livability of rural residents’ living space under the employment dimension are the guarantee (quantity, technology, and output) and accessibility of arable land, the guarantee and accessibility of surrounding industrial enterprises, and the income of rural residents [32,33].

Consumption activities comprise an important part of the daily activities of rural residents. In addition, the spatial livability of the consumption activity dimension is an important element of the livability level of rural living spaces. The level of livability under the dimension of consumer employment activities refers to the extent to which rural residents’ consumption needs are met in terms of vegetables and food, daily necessities, clothing and accessories, furniture and electrical appliances, and comprehensive shopping [34,35]. We considered the livability of rural residents’ living spaces under the dimension of consumption activities by choosing the number, diversification, and accessibility of consumption places.

Leisure activities are indispensable parts of the increasingly diversified daily activities of rural residents as they become more affluent. Spatial livability under the leisure activity dimension is an important component of the livability level of rural living spaces. The leisure activities of rural residents refer to the sum of their inner experience and behavior, free from the shackles of the outside world and free pursuit of happiness, satisfaction, physical and mental pleasure, and self-development. In this case, the level of daily activities of rural residents is high [36]. With the improvement in people’s living standards, the participation of rural residents in sports leisure, outdoor recreation, recreation and leisure, tourism leisure, and cultural leisure activities has become increasingly frequent. In this context, we considered the habitability of leisure activities and selected indicators, such as the number, diversification, and accessibility of leisure places.

### 2.4. Methods

#### 2.4.1. The Evaluation Index System

Based on the aforementioned analysis framework, with scientificity and availability as the basic principles of our evaluation dimension selection, and on the basis of the living activities of rural residents falling under the four categories (i.e., residence, employment, consumption, and leisure), we constructed an evaluation index system of the livability level of rural living spaces in Jianghan Plain consisting of 36 indicators under 4 dimensions. These dimensions were as follows: residential activity, employment activity, consumer activity, and leisure activity.

#### 2.4.2. Computation of Indices

(1) Calculation of accessibility index

The data were preprocessed before reachability calculation. In this study, accessibility was defined as the time cost of the nearest facility in rural residential plaques within the county area [37]. In accordance with the road design regulations and traffic laws of China, the speed of each type of road was set, the vector data of all types of roads were converted into raster data, and the time spent for 1 km was selected as the time cost value of the raster. We used ArcGIS to merge the cost layers of various roads, follow the principle of minimizing the cost of each grid, and finally obtain the traffic raster cost layer (Table 2). By using the cost distance analysis tool of ArcGIS, we calculated the minimum cumulative cost distance and direction from a rural residence to its nearest location of education, medical treatment, cultivated land, and various consumption and leisure. Then, we used ArcGIS cost path analysis tools to calculate rural areas separately and the shortest path from a house to its nearest education, medical, arable land, and various consumption and leisure locations. The accessibility values of various indicators were obtained by calculating average values by county.

(2) Calculation of diversification indicators

The degree of diversification was measured using the Shannon–Weiner index. The higher the index, the greater the difference in consumption or leisure activities provided by regions, and the richer the types of facilities [38].

#### 2.4.3. Livability Level Evaluation Method

The data were processed using the “minimum–maximum criterion method” to eliminate dimensional effects among indexes. The corresponding weight of each index was calculated using the entropy method. Then, the livability level of rural living spaces in Jianghan Plain was calculated using the linear weighting sum method. The objectives were as follows: (1) to overcome the problems of randomness and subjective judgment that cannot be avoided by the subjective weighting method, and (2) to solve the problem of overlapping information among multi-indicator variables. The details of the formulas and procedures used can be found in [39,40,41]. Table 3 presents the weight of each indicator. The formula is as follows:

$$p_{\mathrm{ij}}=\frac{X_{\mathrm{ij}}^{\prime }}{\sum_{i=1}^{m}X_{\mathrm{ij}}}\;e_{j}=-\frac{1}{\mathrm{In}m}\sum_{i=1}^{m}p_{\mathrm{ij}}{\mathrm{In}(p}_{\mathrm{ij}})$$

where $p_{\mathrm{ij}}$ is the proportion of index j in region i; $X_{\mathrm{ij}}^{\prime }$ is the standardized matrix; $X_{\mathrm{ij}}$ is the original matrix; $e_{j}$ is the entropy value, with 0 ≤ $e_{j}$ ≤ 1; and m is the number of county-level administrative districts in the Jianghan Plain, m = 20.

$$q_{j}=1-e_{j}\;w_{j}=\frac{q_{j}}{\sum_{i=1}^{k}q_{j}}$$

where $q_{j}$ is the standard coefficient; $w_{j}$ is the weight of each indicator; and *k* is the number of evaluation indicators.

$$Z_{i}=\sum_{j=1}^{k}w_{j}\times p_{\mathrm{ij}}$$

where $Z_{i}$ is the score of the livability of rural living spaces in region *i*.

## 3. Results

### 3.1. Overall Characteristics of the Livability Level of Rural Living Spaces

The livability of rural living spaces in Jianghan Plain is evidently characterized by “grade difference”, and the overall livability level is not high.

Under the aforementioned framework and method, the average livability score of rural living spaces in 20 counties in Jianghan Plain was calculated. The total score of the livability of rural living spaces in Jianghan Plain was 0.4285, and the coefficient of variation was 27.7022%. The score was less than 0.5, indicating that the overall livability level of rural living spaces at the county level in Jianghan Plain must be improved. Certain regional differences existed in the livability level of rural living spaces in the county. The difference between high and low grades was evident. The lowest score (0.2940) was obtained for Jiangling, whereas the highest score (0.7782) was reported in Jingzhou, i.e., 2.6466 times the score of Jiangling.

**Figure 3:**
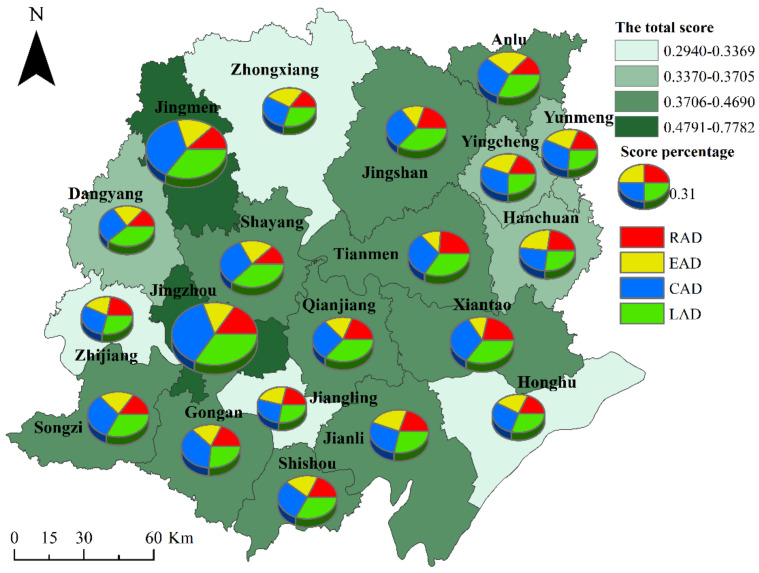
Spatial distribution pattern of livability level of rural living spaces in Jianghan Plain.

To fully present the spatial pattern, the natural breakpoint method was used. It involves dividing into one to four levels in accordance with the score from highest to lowest. With the help of ArcGIS software (Eari Corporation, Redlands, CA, USA), the scores of each research unit were spatially correlated with each research unit in vector format, drawing the Jianghan Plain rural living space livability spatial distribution map. Figure 3 shows the spatial distribution of the total score of the livability of rural living spaces in each county-level administrative unit in the Jianghan Plain and the pie chart of the score percentage of the livability of rural living spaces in the dimension of residential, employment, consumption and leisure activities. The average livability of rural living spaces in high-livability counties was 2.3196 times the average in low-livability counties, and the difference between groups was also apparent. A statistical analysis of the number of counties at each level showed that the number of counties with high livability was two, which is a relatively low percentage. Meanwhile, the number of counties in the higher livability categories was high, accounting for 50% of the total number of cities. The number of counties in the lower livability categories and counties with low livability was equal, i.e., both were four, and accounted for 20% of the total number of counties.

High-grade rural living space livability areas were Jingzhou and Jingmen, with an average value of 0.7405. Ten counties were at a higher level, with an average value of 0.4352, while half of the counties had relatively high-grade rural living space livability. The livability scores of rural living spaces in Hanchuan, Dangyang, Yunmeng, and Yingcheng were relatively low, with an average value of 0.3649. The low-grade rural living space livability areas were Shayang, Honghu, Zhijiang, and Jiangling, with an average score of 0.3192. These two areas require more attention.

### 3.2. Characteristics Analysis of the Subdimensions

The livability scores of all the dimensions of rural living spaces in Jianghan Plain were arranged as follows: “residence < employment < consumption < leisure”.

In terms of subdimension scores, the average score of the livability of the residential activity dimension in Jianghan Plain was the lowest among the four dimensions, with an average score of 0.0748. The living space livability level in the employment activity dimension was the second, with an average score of 0.0874. The livability level of living spaces in the dimension of consumption activities was slightly lower than that of leisure activities. The average scores of the livability level of living spaces in Jianghan Plain in the dimensions of consumption and leisure activities were 0.1278 and 0.1386, respectively.

The higher the livability level of rural living spaces in Jianghan Plain, the larger the difference between the scores of the subdimensions.

**Figure 4:**
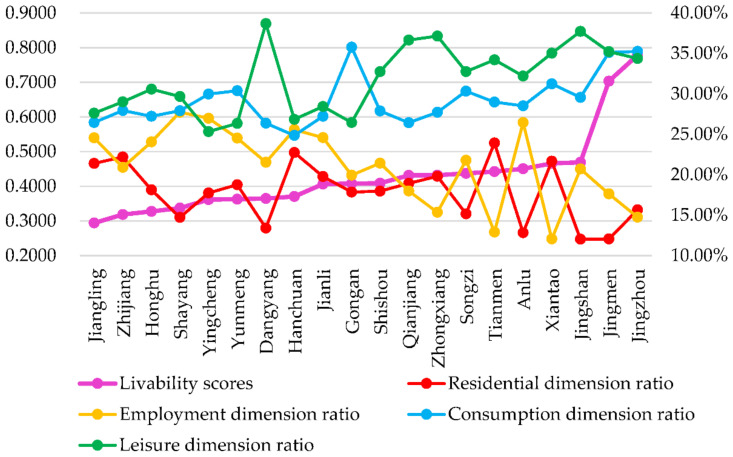
Livability scores and percentage of livability scores of subdimensions in Jianghan Plain.

Figure 4 shows a line graph of livability scores and the percentage of livability scores of subdimensions in Jianghan Plain. In terms of livability scores and the percentage of livability scores of subdimensions, the higher the scores, the smaller the percentage of living spaces in the dimensions of residence and employment. Moreover, the percentages of livability scores of living spaces in the consumption and leisure dimensions were larger. This result indicates that rural residents seek diversity in their lives with the development of the rural economy and improvement in rural life. Improving the overall livability level of rural residents’ living spaces will not only improve the livability level of rural living spaces in all the dimensions of rural residents but will also increase the proportion of rural residents with consumption and leisure needs that must be met. Thus, an important guarantee of high-level daily activities is available for rural residents.

### 3.3. Problem Area Identification

The identification of problematic areas and the lower categories of corresponding indicators in problem areas can guide governments and builders to clarify the key direction of improvement in the livability of their rural living spaces. On the basis of the livability level index of rural living spaces in Jianghan Plain in 2018, problem areas were identified based on an index that was less than or equal to the “average minus standard deviation”. If one of the indexes of a county was less than or equal to the “average minus standard deviation”, then it was defined as the problem area [42]. The results showed that the number of counties with low livability levels of rural living space in the consumption and leisure dimensions was all one. Two counties had low livability of rural living space in the dimension of consumption. Four counties had low livability of rural living space in the residential dimension. Finally, three categories of seven problem counties were identified. Thereafter, problem indicators were calculated for the problem areas.

(1) The low residential dimension areas included Dangyang and Shayang.

Problem indicators in Dangyang were the comfort level of residence and income status of rural residents.

Problem indicators in Shayang were the comfort level and accessibility of residence.

(2) The low employment dimension areas included Zhijiang, Zhongxiang, Xiantao, and Tianmen.

The problematic indicator in Zhijiang was the income status of rural residents.

The problem indicator in Zhongxiang was the accessibility of arable land.

The problematic indicators of Xiantao were the accessibility of educational and medical facilities around a residence, arable land, industrial enterprises, consumer places, and leisure places.

The problem indicator in Tianmen was the guarantee of arable land.

(3) The low consumption–leisure dimension area included Jiangling.

The problem indicators in Jiangling were the guarantee of the security of educational and medical facilities around a residence, the guarantee of arable land, the guarantee of industrial enterprises, the number of consumer places, and the diversification of leisure places.

## 4. Discussion

### 4.1. Framework for Assessing the Livability Level of Rural Living Space

In recent decades, the livability of living space has attracted increasing attention. Previous research has focused on the evaluations of urban livability and the construction of livable cities [13]. Studies on the livability of living space in rural areas are relatively few, and systematic research on the livability of rural living spaces remains weak and limited. Rural areas are the spatial carriers for rural residents to conduct various daily activities. The livability level of rural living spaces is the ability level to meet the major living activities of residents in their region, such as residence, employment, consumption, and leisure. For the first time, the current research was performed starting from the daily residence, employment, consumption, and leisure activities of rural residents. Then, we constructed a research framework for the analysis of the livability of rural living spaces from the four aspects of livability: residence, employment, consumption, and leisure. Setting the county area as the basic unit of research and Jianghan Plain as the case site, we obtained the land use, POI, socioeconomic, road, and other data to quantitatively evaluate the livability of rural living spaces. In this study, an entropy method was used, which is more objective than using qualitative methods to obtain index weights and comprehensive indexes [43]. To a certain extent, this study enriches the conceptual content of the livability of rural living spaces and can provide further insight for similar research in the future.

### 4.2. Improvement in Rural Living Space Livability Level 

The direct purpose of building livable rural living spaces is to meet the needs of residents [20]. The evaluation, monitoring, and improvement of the livability of rural living spaces are highly significant to the construction of rural areas and improvement in the living standards of rural residents. This study found that the livability level of rural living spaces varied among the regions in Jianghan Plain, with wide disparities between them, and livability must be improved. In terms of the four dimensions of the livability of rural living spaces examined in this study, the livability levels of consumption and leisure were relatively high, whereas those of residential and employment were relatively low. This result showed that the living and employment conditions in the daily life of rural residents must still be improved. This case reminds local government agencies to be more targeted in their livability enhancement efforts for rural living spaces. In particular, this study argued that the improvement in the livability of rural residents’ living spaces can be based on the satisfaction of rural residents’ daily life needs. Then, we determined how to optimize the livability of rural living spaces in the study area. In accordance with the spatial differences in the livability levels of rural living spaces in Jianghan Plain, the livability of living space in Jianghan Plain can be optimally regulated as follows: (1) Improve county economic development and the income level of rural residents to provide a material basis for improving the livability of rural residents’ living space. (2) Optimize county road infrastructure, improve road traffic environment in rural areas, improve the accessibility of residence, and enhance the accessibility of agricultural land, surrounding factories, consumption places, and leisure activity places to implement the overall improvement of the livability of rural residents’ living spaces. (3) Pursue the construction of new rural communities; improve the comfort level of rural residents’ life in Dangyang, Jingshan, Shayangdents, and other counties; and further enhance the livability level of rural residents’ living spaces in the residential activity dimension. (4) Pursue the construction of attractive villages, improve the supply service capacity for rural residents’ consumption and leisure needs in combination with the preparation for rural planning, and gradually improve the livability level of living space in the region.

### 4.3. Limitations and Further Research

However, some limitations exist in this study. First, the data currently used were at the county level, which disregarded the livability differences of rural living spaces within a county and did not study the livability of rural living spaces at the individual level. Second, limited by the availability of indicators, the indicator system in this study may not be comprehensive. Moreover, this system should be enriched by using long-term data and including more data types. Third, this study only quantitatively evaluated the livability level of rural living spaces in Jianghan Plain and did not study the reasons for the formation of regional differences and the policy background behind the differences. In the future, we will further study the reasons that affect the level of livability and the factors that influence the differences in the livability of rural living spaces.

## 5. Conclusions

In this study, we proposed a new evaluation framework based on the components of the major daily activities of rural residents and conducted a case study on the spatial patterns of rural living spaces in China’s Jianghan Plain. The major findings can be summarized as follows: First, a residence is the most basic need in life, employment provides the most basic material guarantee for the survival of residents, consumption is an important part of the daily activities of rural residents, and leisure activities are necessary and indispensable part of life activities. The evaluation analysis of the livability level of rural living spaces from the perspective of the daily activities of rural residents enriches the connotation of rural livability and related studies. Second, this study showed that the comprehensive use of diverse data, such as land use, POI, socioeconomic characteristics, administrative boundary, and road data, can enrich the research level of the spatial patterns associated with the livability of rural living spaces. It can also provide data support for improvement in rural living space livability in the Jianghan Plain region of China and a reference for the analysis of rural living space livability in other similar traditionally agricultural areas. Finally, within the context of rural revitalization strategy, policymakers should not only continuously improve the level of the overall economic development of rural areas and improve the hard environment infrastructure such as roads and other basic guarantees but also pay attention to the convenience of the daily activities of rural residents and the degree of satisfaction they derive from diversified needs of their daily life. Rural construction is carried out on the basis of studying the livability of rural living spaces, thus promoting the attractiveness of rural areas.

Improving the livability of rural living spaces is an important part of the Chinese government’s work to improve the living environment of rural residents. With this study, we hope to inspire future researchers who are interested in the daily activities of rural residents and the livability of rural living spaces. The study also provides a reasonable basis for the establishment of a more scientific, accurate, and comprehensive index system in the future. Furthermore, this study provides a more targeted and constructive reference for the development of the livability of rural living spaces from the perspective of the daily activities of rural residents.

## Figures and Tables

**Table 1:** Point of interest types and classifications.

POI Types	POI Classification	Quantity
Educational facilities	Elementary schools, middle schools, etc.	2296
Medical facilities	Clinics, specialty hospitals, general hospitals	2389
Factories	Factories	2292
Vegetable food consumption	Fruit markets, agricultural products, sideline products markets, vegetable markets, fish and seafood markets	1227
Consumption for daily necessities	Convenience supermarkets, convenience stores, small commodity markets	8665
Clothing accessories consumption	Clothing, shoes, hats, and leather goods stores, shopping malls	8765
Furniture, electrical appliances consumption	Home appliance and electronics stores, home building material markets	10,842
Comprehensive shopping consumption	Super supermarket, comprehensive market, shopping place	7921
Sports leisure	Sports complexes	167
Outdoor recreation	Picking gardens, fishing gardens	247
Tourism leisure	Tourist attractions	1473
Recreation and leisure	Singing rooms, bars, chess and card rooms, game halls, amusement parks, Internet cafes, movie theaters	2442
Cultural leisure	Museums, zoos, botanical gardens	33

**Table 2:** Relative time cost settings of different road types.

	Railways	Highways	National Roads	Provincial Roads	County Roads	Township Roads	Water	Others
Speed (km/h)	150	100	70	50	30	25	-	-
The time cost (h)	0.4	0.6	0.86	1.2	2	2.4	3	30

**Table 3:** Evaluation index system of the livability level of rural living spaces.

Dimensions	Indicators	Weight
Residential activity dimension (RAD)	Rural residential area per capita ^1^	0.0258
	Degree of greening around a residence ^2^	0.0153
	Road length per unit area ^3^	0.0216
	Number of educational facilities	0.0327
	Number of medical facilities	0.0380
	Accessibility of educational facilities	0.0153
	Accessibility of medical facilities	0.0146
Employment activity dimension (EAD)	Per capita arable land area	0.0180
	Agricultural technology level ^4^	0.0282
	Efficiency of agricultural output value ^5^	0.0281
	Accessibility of arable land	0.0183
	Number of industrial enterprises	0.0496
	Accessibility of industrial enterprises	0.0146
	Per capita disposable income of rural residents	0.0214
Consumption activity dimension (CAD)	Number of vegetable food consumption places	0.0732
	Number of consumer places for daily necessities	0.0421
	Number of clothing and accessory consumption places	0.0435
	Number of furniture and electrical appliance consumption places	0.0379
	Number of comprehensive shopping and consumption places	0.0337
	Diversification of consumption places	0.0080
	Accessibility of vegetable food consumption places	0.0147
	Accessibility of consumption places for daily necessities	0.0145
	Accessibility of clothing and accessory consumption places	0.0149
	Accessibility of furniture and electrical appliance consumption places	0.0146
	Accessibility of comprehensive shopping and consumption places	0.0149
Leisure activity dimension (LAD)	Number of places for sports leisure activities	0.0644
	Number of places for outdoor recreation activities	0.0404
	Number of places for recreation and leisure activities	0.0642
	Number of places for tourism leisure activities	0.0269
	Number of places for cultural leisure activities	0.0479
	Diversification of leisure places	0.0291
	Accessibility of sports leisure places	0.0147
	Accessibility of outdoor recreation places	0.0147
	Accessibility of recreation and leisure places	0.0147
	Accessibility of tourism leisure places	0.0149
	Accessibility of cultural leisure places	0.0149

^1^ Rural residential area per capita = rural residential area/rural population. ^2^ Degree of greening around a residence = fertilizer application/arable land area. ^3^ Road length per unit area = total road length/county unit area. ^4^ Agricultural technology level = total grain output/grain sown area. ^5^ Efficiency of agricultural output value = total agricultural output value/crop sown area.

## Data Availability

Not applicable.

## References

[1] Zhan K., Song S. (2003). Rural—urban migration and urbanization in China: Evidence from time-series and cross-section analyses. China Econ. Rev..

[2] Han D., Qiao J., Zhu Q. (2021). Rural-spatial restructuring promoted by land-use transitions: A case study of Zhulin Town in central China. Land.

[3] Liu Y. (2020). The basic theory and methodology of rural revitalization planning in China. Acta Geogr. Sin..

[4] Hu S., Yu B., Wang M. (2019). Rural restructuring and transformation: Western experience and its enlightenment to China. Geogr. Res..

[5] The CPC Central Committee and the State Council Issued the 2018–2022 Strategic Planning for Revitalization of Rural Areas. http://www.gov.cn/xinwen/2018-09/26/content_5325534.htm.

[6] Wang Y., Zhu Y., Yu M. (2019). Evaluation and determinants of satisfaction with rural livability in China’s less-developed eastern areas: A case study of Xianju County in Zhejiang Province. Ecol. Indic..

[7] Xia R., Wu Y., Qian J. (2019). A review of domestic rural livability evaluation studies based on bibliometrics. Jiangxi Agric..

[8] Tomida M., Hoshino K., Tomida M., Hoshino K. (1985). An Introduction to Rural Geography.

[9] McGrath B. (1998). The sustainability of a car dependent settlement pattern: An evaluation of new rural settlement in Ireland. Environmentalist.

[10] Zhang Z., Ju J., Chen Z. (2014). The livable evaluation and analysis of characteristics of space in Lanzhou. Acta Geogr. Sin..

[11] Zhang Y., Fei Y., Ge Q. (2016). Analysis of living space based on BDI decision: A case study for Dalian Shahekou district. Geogr. Res..

[12] Zhan D., Zhang W., Dang Y., Qi W., Liu Q. (2017). Urban livability perception of migrants in China and its effects on settlement intention. Prog. Geogr..

[13] Li Y., Qiao L., Wang Q., Karácsonyi D. (2020). Towards the evaluation of rural livability in China: Theoretical framework and empirical case study. Habitat Int..

[14] Gao L., Li H., Zhang X. (2020). Historical development and prospect of rural living space research in China. Prog. Geogr..

[15] Yu B., Lu Y., Zeng J., Zhu Y. (2017). Progress and prospect on rural living space. Sci. Geogr. Sin..

[16] Wu M. Strategies for optimizing rural living space under the change of residents’ lifestyles: The case of Yicheng City, Hubei Province. Proceedings of the Annual National Planning Conference.

[17] Feng W., Zhou W., Li A., Zhang B. (2008). GIS-based spatial analysis on rural settlement centralization in the upper minjiang river basin: A case study of Maoxian County. Resour. Environ. Yangtze Basin..

[18] Xiao F., Du Y., Ling F., Gao A., Li Y. (2012). Spatial pattern of villages in Jianghan plain and its relationships with the micro-topography. Geogr. Res..

[19] Li Y., Tian G. (2015). The cognitive analysis of city space livability based on semantic differential. Henan Sci..

[20] Hu Q., Wang C. (2020). Quality evaluation and division of regional types of rural human settlements in China. Habitat Int..

[21] Tang G., Zhao M. (2000). Study on spatial distribution law of rural settlements based on GIS—taking Yulin area in northern Shaanxi as an example. Econ. Geogr..

[22] Li B., Zeng J. (2009). Research on rural human settlement environment based on the changes of the householders’ spatial behaviors. Geogr. Geo-Inf. Sci..

[23] Geographical Information Monitor Cloud Platform. http://www.dsac.cn/DataProduct/Detail/20080416.

[24] National Geomatics Center of China http://www.ngcc.cn/ngcc.

[25] Open Street Map. https://www.openstreetmap.org.

[26] Liao W., Sun M., Yu C., Deng Y., Li M., Yang J., Li Y., Xu J., Chen Y., Yan Y. (2021). Impact Factors of COVID-19 Epidemic Spread in Hubei Province Based on Multi-Source Data. Trop. Geogr..

[27] Zong W., Cheng L., Xia N., Jiang P., Wei X., Zhang F., Jin B., Zhou J., Li M. (2018). New technical framework for assessing the spatial pattern of land development in Yunnan Province, China: A “production-life-ecology” perspective. Habitat Int..

[28] Zhang H. (2020). A Study on the Livable Character of Rural Living Space and Its Influencing Factors in Jianghan Plain. Master’s Thesis.

[29] Li X., Yang H., Jia J., Shen Y., Liu J. (2021). Index system of sustainable rural development based on the concept of ecological livability. Environ. Impact Asses. Rev..

[30] Li H., Zhang X., Wu J., Wu Q. (2012). Spatial pattern and influencing factors of settlement in less developed areas: Taking Suzhou of Anhui province as an example. Sci. Geogr. Sin..

[31] Woods M. (1997). Discourses of power and rurality—Local politics in somerset in the 20th century. Political Geogr..

[32] Hoskins W. (1955). The Making of the English Landscape.

[33] Kiss E. (2000). Rural restructuring in hungary in the period of socio-economic transition. GeoJournal.

[34] Jiu J., Zhang M. (2016). Daily Consumption Activities of Different Classes Impacted by Smog and Their Constraint. Trop. Geogr..

[35] Li B., Liu P., Zhang B., Tian Y. (2011). The evolution of spatial structure of rural households’ consuming behaviors in undeveloped rural areas and its influencing factors: A case of Ercheng Town in Hubei Province. Prog. Geogr..

[36] Guo X., Yu B., Zhuo R., Li R., Zeng J. (2020). Research on the changes and influencing factors of farm housholds’ daily leisure in Jianghan plain. Hum. Geogr..

[37] Yin J., Wang X., Jia Y., Li C., Wang J. (2019). Spatial accessibility distribution characteristics and influencing factors of the tourism poverty alleviation key villages in Wuling Mountain area, Hubei Province. Prog. Geogr..

[38] Shan R., Zhang S. (2021). The vitality assessment of renewed historic built environment and a discussion on regeneration strategies: The cases of Tianzifang, Xintiandi, and Yuyuan Tourist Mall in Shanghai. Urb. Plan. Forum.

[39] Wang Y., Sarkar A., Ma L., Wu Q., Wei F. (2021). Measurement of Investment Potential and Spatial Distribution of Arable Land among Countries within the “Belt and Road Initiative”. Agriculture.

[40] Liu Q., Wang S., Zhang W., Li J., Zhao Y., Li W. (2017). China’s municipal public infrastructure: Estimating construction levels and investment efficiency using the entropy method and a DEA model. Habitat Int..

[41] Chen Y., Miao Q., Zhou Q. (2022). Spatiotemporal Differentiation and Driving Force Analysis of the High-Quality Development of Urban Agglomerations along the Yellow River Basin. Int. J. Environ. Res. Public Health.

[42] Tu S., Zheng Y., Long H., Wan S., Liang X., Wang W. (2020). Spatio-temporal pattern of rural development and restructuring and regional path of rural vitalization in Guangxi, China. Acta Geogr. Sin..

[43] Zeng X., Fu Z., Deng X., Xu D. (2021). The Impact of Livelihood Risk on Farmers of Different Poverty Types: Based on the Study of Typical Areas in Sichuan Province. Agriculture.

